# Effects of Hydrogen Peroxide Soaking on the Seeds of Different Edible Bean Varieties

**DOI:** 10.3390/plants14223476

**Published:** 2025-11-14

**Authors:** Ruili Dong, Zexiang Gao, Yapeng Gao, Junchi Tang, Xuguang Shen, Xin Ding, Chao Ma, Chunxia Li

**Affiliations:** Agronomy College, Henan University of Science and Technology, Luoyang 471000, China; d18339655702@163.com (R.D.); g17716595202@163.com (Z.G.); 15703846924@163.com (Y.G.); tangjc626@gmail.com (J.T.); 13939231256@163.com (X.S.); 16639108071@163.com (X.D.); machao840508@163.com (C.M.)

**Keywords:** hydrogen peroxide, soaking, antioxidant enzymes, storage of substances

## Abstract

To clarify the effects of hydrogen peroxide (H_2_O_2_) seed soaking on the germination and stress resistance of different edible bean seeds, seeds of mung bean (*Vigna radiata* L. ‘Keda Green No. 2’), cowpea (*Vigna unguiculata* L. ‘Keda Cowpea No. 1’), and red bean (*Vigna umbellata Thunb.* ‘Jihong 352’) were soaked in a 50 mmol/L H_2_O_2_ solution. The study examined the germination and growth-related physiological indices of seeds after soaking. The results showed that hydrogen-peroxide-primed seeds of mung bean (GBH), cowpea (CBH), and red bean (RBH) exhibited significant improvements in germination performance and physiological activity compared with their respective controls (GBCK, CBCK, and RBCK). The results indicated that H_2_O_2_ soaking significantly improved the germination ability of the seeds, with the germination rate of mung beans, cowpeas, and red beans increasing by 48.89%, 21.11%, and 18.89%, respectively, and the germination percentage increasing by 31.11%, 24.45%, and 17.77%. Additionally, H_2_O_2_ soaking enhanced the activity of α-amylase, protease, and the antioxidant enzymes peroxidase (POD), superoxide dismutase (SOD), and catalase (CAT); increased the soluble sugar and soluble protein content in the seeds; and reduced the malondialdehyde (MDA) content. The experiment demonstrated that H_2_O_2_ promotes the germination of mung bean, cowpea, and red bean seeds by influencing antioxidant enzyme activity, the breakdown of storage substances, and the regulation of germination-related substances, thereby improving seedling adaptation to environmental stress. This study aims to improve the germination rate of legume seeds using H_2_O_2_ treatment, providing a theoretical basis for techniques to enhance seed vigor, especially for seeds that perform poorly in germination under normal conditions.

## 1. Introduction

Globally, legumes are one of the most important food crops, widely distributed across various regions, and they play a significant role in global agriculture and the food industry [1,2,3]. Legume crops not only serve as an important source of protein for humans and animals, but also play an indispensable role in agricultural ecosystems [4,5]. Especially in dryland areas, legumes, due to their strong drought resistance and nitrogen-fixing capabilities, have become important autumn crops and economic crops. However, with the intensification of global climate change, agricultural production in dryland regions faces increasingly severe challenges, particularly the scarcity of water resources, which has become one of the key limiting factors for crop growth [6]. In these regions, insufficient soil moisture makes it difficult for seeds to absorb enough water to initiate the germination process, leading to significant physiological defects during crop growth and development. At the same time, poor soil fertility and adverse environmental conditions (such as extreme temperatures and drought) severely affect seed germination percentage and seedling growth [7]. These negative factors, especially during the germination and seedling stages, have a growing impact on crop growth, directly limiting production potential. Therefore, improving seed germination ability and seedling stress resistance has become a critical issue in dryland agricultural production.

During seed germination, the breakdown of stored macromolecular nutrients within seeds provides energy for germination and seedling growth, thereby influencing seedling development [8]. During the early stages of germination, the expression of α-amylase-related genes is upregulated, and α-amylase activity increases, promoting the conversion of starch into soluble sugars, which provide energy for seed germination and seedling growth [9]. At the initial stages of germination, soluble protein content increases, regulating osmotic potential, enhancing the seed’s water absorption ability, and increasing the moisture content within the seed. When the soluble protein reaches a certain level, it begins to hydrolyze into amino acids, providing a major nitrogen source for the growth of the seed embryo [10]. During the process of seed germination, in addition to the degradation of starch, the hydrolysis of storage proteins is equally crucial for embryo development. Proteases play a key role in this process by catalyzing the breakdown of storage proteins into amino acids and small peptides, which provide a nitrogen source for the synthesis of new proteins required for cell division and differentiation. Therefore, protease activity is regarded as an important indicator of seed metabolic activation. When plant organs age or the plant is damaged under stress conditions, membrane lipid peroxidation often occurs, and malondialdehyde (MDA) is the final product of membrane lipid peroxidation [11]. Therefore, measuring MDA content in plants can reflect the extent of damage to the cell membrane, thus indicating the plant’s ability to adapt to stress [12]. Peroxidase (POD), catalase (CAT), and superoxide dismutase (SOD) are key enzymes in the plant antioxidant system, and their activity levels reflect the extent of external stress the plant is experiencing. Under normal conditions, plants generate reactive oxygen species (ROS) during metabolic processes, which are highly oxidative substances. In plants, ROS are maintained in a dynamic balance, with generation and clearance rates being similar, preventing excessive accumulation that could harm the plant [13]. POD, CAT, and SOD are the three main enzymes responsible for scavenging ROS. Under stress conditions, the production in ROS increases significantly, and the activity of these three enzymes also increases accordingly to clear the ROS, maintaining normal plant metabolic processes and protecting plants from damage. Thus, the activity of POD, CAT, and SOD is an important indicator of plant stress resistance [14]. The process of seed germination is highly complex and involves multiple physiological events, such as water absorption, protein hydration, subcellular structural changes, increased respiration rates, macromolecule synthesis, cell elongation, and division. These processes must be coordinated under certain environmental conditions, including water, temperature, light, and oxygen. However, under certain adverse environmental conditions, seeds may enter a dormant state, leading to germination disorders. In such cases, breaking the dormancy and activating related physiological processes are necessary to promote seed germination.

With the development of agricultural technologies, seed priming is now considered an effective strategy to improve germination and enhance stress resistance [11]. Among them, hydrogen peroxide (H_2_O_2_), a plant growth regulator with broad application potential, has attracted significant attention from both the academic and agricultural communities in recent years. As a strong oxidizing agent, H_2_O_2_ is not only an important signaling molecule in plants but also plays a critical role in various physiological processes [15]. H_2_O_2_ can regulate the redox state of plant cells, and participate in plant growth and development, stress responses, seed germination, and other physiological processes. Whether endogenous or exogenous, H_2_O_2_ can enhance plant stress tolerance by regulating the antioxidant system and improving water and nutrient absorption [16,17].

However, although many studies have explored the effects of H_2_O_2_ treatment on crop seed germination, research on different legume crops (such as mung beans, cowpeas, red beans, etc.) remains limited, especially regarding the differences in response to H_2_O_2_ treatment and the underlying physiological mechanisms. Therefore, this study aims to treat mung bean, cowpea, and red bean seeds with a 50 mmol·L^−1^ H_2_O_2_ solution and analyze their germination percentage, germination energy percentage, soluble sugar, soluble protein, antioxidant enzyme activity, α-amylase activity, protease activity, and MDA content. The goal is to evaluate the effects of H_2_O_2_ on seed germination and stress resistance in these legumes. This research provides theoretical guidance for improving post-planting emergence rates and seedling growth in mung beans, cowpeas, and red beans, as well as scientific support for the high-yield, efficient cultivation of legume crops in dryland areas.

## 2. Result

### 2.1. Effects of H_2_O_2_ Soaking on Germination Capacity

Seed germination is a critical phase in the early life of plants, serving not only as the starting point of plant reproduction but also as the foundation for the continuation of species. In this study, compared to the control group (Table 1), H_2_O_2_ seed soaking significantly improved the germination percentage, germination energy percentage, germination vigor percentage, and germination index of mung beans, cowpeas, and red beans. After H_2_O_2_ treatment, mung beans’ germination energy percentage increased by 48.89%, their germination percentage increased by 31.11%, and their germination index increased by 27.66%, compared to the control group. For cowpeas, the germination vigor percentage increased by 19.18%, the germination energy percentage increased by 158.36%, the germination percentage increased by 33.85%, and the germination index increased by 38.94%, compared to the control. For red beans, the germination vigor percentage increased by 23.19%, the germination energy percentage increased by 18.89%, the germination percentage increased by 23.52%, and the germination index increased by 91.67%. The differences between the H_2_O_2_ treatment and the control group for all four indices were significant, indicating that H_2_O_2_ soaking can significantly enhance the germination capacity of mung beans, cowpeas, and red beans.

### 2.2. Effects of H_2_O_2_ Soaking on Seedling Growth

**Figure 1:**
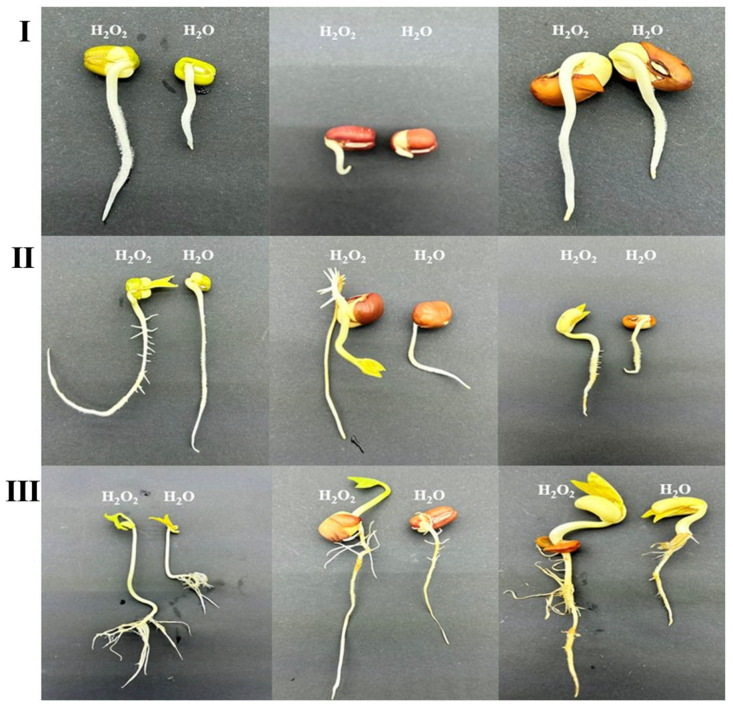
Changes in seeding and growth of mung beans, red beans, and cowpeas after H_2_O_2_ soaking. Note: The images depict, in left-to-right order, mung beans, red beans, and cowpeas. (**I**): Changes in the germination and growth of mung beans, red beans, and cowpeas 24 h after the end of H_2_O_2_ soaking. (**II**): Changes in the germination and growth of mung beans, red beans, and cowpeas 72 h after the end of H_2_O_2_ soaking. (**III**): Changes in the germination and growth of mung beans, red beans, and cowpeas at 120 h after the end of H_2_O_2_ soaking.

As shown in Table 2, the radicle lengths of mung bean, cowpea, and red bean seeds in the H_2_O_2_ soaking treatment group were significantly longer than those in the control group at 24 h, 48 h, and 72 h after germination. Specifically, the radicle lengths of mung beans increased by 46.87%, 37.78%, and 26.02%, respectively; cowpeas increased by 25.00%, 26.19%, and 26.92%, respectively; and red beans increased by 40.00%, 20.00%, and 20.93%, respectively. The growth status of the treated seeds was notably faster than that of the untreated controls, with cotyledons developing more rapidly (Figure 1). These results indicate that H_2_O_2_ treatment can effectively promote the germination and growth of mung bean, cowpea, and red bean seeds.

### 2.3. Effects of H_2_O_2_ Soaking on Soluble Sugar Content During Germination

**Figure 2:**
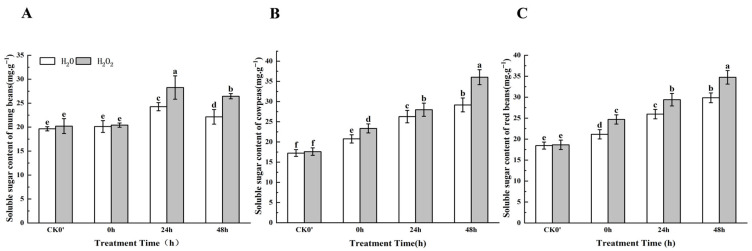
The soluble sugar content in different legumes under H_2_O_2_ and water seed soaking treatment. (**A**) Soluble sugar content of mung beans. (**B**) Soluble sugar content of cowpeas. (**C**) Soluble sugar content of red beans. CK0′ represents the seeds at the end of soaking; “0 h” represents the seeds whose radicle has just broken through the seed coat (germination 0 h); “24 h” refers to seed samples which had been cultured for 24 h after germination; “48 h” refers to seed samples which had been cultured for 48 h after germination. The different letters mean significant differences at *p* < 0.05.

During 0–48 h after germination, the soluble sugar content showed an increasing trend in both the control and treated seeds, but the increase was more pronounced in seeds treated with H_2_O_2_. (Figure 2). Specifically, for mung beans, the soluble sugar content increased by 1.40%, 14.15%, and 16.25% at 0 h, 24 h, and 48 h, respectively. At the 24 h and 48 h time points, the content of soluble sugars after H_2_O_2_ treatment showed significant differences compared with the control. In cowpeas, the soluble sugar content increased significantly by 13.59%, 6.08%, and 19.05% at 0 h, 24 h, and 48 h, respectively. In red beans, the soluble sugar content increased significantly by 17.00%, 11.73%, and 19.83% at the same time points. The results indicate that the effect of H_2_O_2_ on the soluble sugar content in mung beans, cowpeas, and red beans was most significant after 24 h of germination.

### 2.4. Effect of H_2_O_2_ Seed Soaking on α-Amylase Activity During Seed Germination

**Figure 3:**
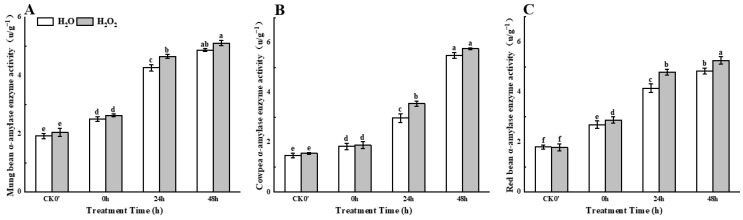
The α-amylase activity in different legumes under H_2_O_2_ and water seed soaking treatment. (**A**) α-amylase activity of mung beans. (**B**) α-amylase activity of cowpeas. (**C**) α-amylase activity of red beans. CK0′ represents the seeds at the end of soaking; “0 h” represents the seeds whose radicle has just broken through the seed coat (germination 0 h); “24 h” refers to seed samples which had been cultured for 24 h after germination; “48 h” refers to seed samples which had been cultured for 48 h after germination. The different letters mean significant differences at *p* < 0.05.

With the extension of germination time, compared with the control group, H_2_O_2_ seed soaking treatment showed a continuous increase in amylase activity in mung beans, cowpeas, and red beans during 0–48 h after germination (Figure 3). Compared to the control group, the α-amylase activity in mung beans increased by 4.78%, 9.15%, and 4.93% at 0 h, 24 h, and 48 h after germination, respectively, with a significant difference observed at 24 h. In cowpeas, α-amylase activity increased by 2.73%, 19.50%, and 4.93% at 0 h, 24 h, and 48 h, respectively, with a significant difference at 24 h. The α-amylase activity in red beans increased by 7.06%, 15.42%, and 8.90% at 0 h, 24 h, and 48 h, respectively, with a significant difference at 24 h. These results indicate that H_2_O_2_ treatment can enhance α-amylase activity in seedlings.

### 2.5. Effects of H_2_O_2_ Soaking on Soluble Protein Content During Germination

**Figure 4:**
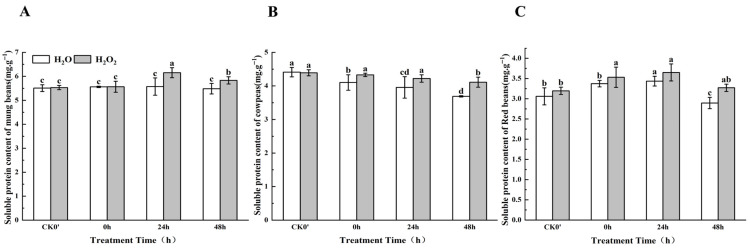
The soluble protein content in different legumes under H_2_O_2_ and water seed soaking treatment. (**A**) Soluble protein content of mung beans. (**B**) Soluble protein content of cowpeas. (**C**) Soluble protein content of red beans. CK0′ represents the seeds at the end of soaking; “0 h” represents the seeds whose radicle has just broken through the seed coat (germination 0 h); “24 h” refers to seed samples which had been cultured for 24 h after germination; “48 h” refers to seed samples which had been cultured for 48 h after germination. The different letters mean significant differences at *p* < 0.05.

Soluble protein content can indirectly reflect the intensity of seed metabolic activity. Over time, the soluble protein content in mung beans and red beans showed an initial increase followed by a decrease during the 0–48 h germination period (Figure 4), whereas cowpeas displayed a gradual decrease in soluble protein content. However, the speed of increase and decrease in soluble protein content between the treatment and control groups differed. Specifically, compared to the control group in mung beans, the soluble protein content increased by 0.11%, 9.37%, and 5.93% at 0 h, 24 h, and 48 h, respectively; the difference between the treated and control seeds was most pronounced at 0 h and 48 h. Compared to the control, H_2_O_2_ soaking led to increases in the soluble protein content of 2.94%, 1.54%, and 5.38% in cowpea seeds at 0 h, 24 h, and 48 h, respectively. In red beans, the soluble protein content increased by 4.46%, 3.10%, and 10.85%, between the treatment group and the control group; this difference was most significant at 0 h and 48 h. These results indicate that H_2_O_2_ treatment can enhance the soluble protein content in seedlings.

### 2.6. Effect of H_2_O_2_ Soaking on Protease Activity During Seed Germination

**Figure 5:**
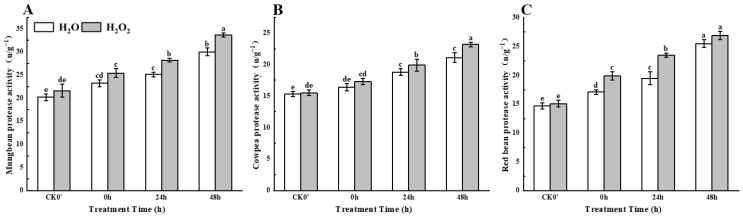
The protease activity in different legumes under H_2_O_2_ and water seed soaking treatment. (**A**) Protease activity of mung beans. (**B**) Protease activity of cowpeas. (**C**) Protease activity of red beans. CK0′ represents the seeds at the end of soaking; “0 h” represents the seeds whose radicle has just broken through the seed coat (germination 0 h); “24 h” refers to seed samples which had been cultured for 24 h after germination; “48 h” refers to seed samples which had been cultured for 48 h after germination. The different letters mean significant differences at *p* < 0.05.

With the passage of time, compared with the control treatment, H_2_O_2_ soaking treatment showed a continuous increase in protease activity in mung beans, cowpeas, and red beans from 0 h to 48 h after germination (Figure 5). Compared to the control, the protease activity in mung beans increased by approximately 9.48%, 12.35%, and 12.33% at 0 h, 24 h, and 48 h of germination, with a significant increase at 24 h. The protease activity in cowpeas increased by 5.48%, 5.85%, and 9.95%, with significant differences between the treatment and control groups at 24 h and 48 h. Protease activity in red beans increased by 16.37%, 20.51%, and 5.49%, with significant differences between the treatment and control groups at 0 h and 48 h. The results indicate that H_2_O_2_ treatment can enhance protease activity in seedlings.

### 2.7. Effects of H_2_O_2_ Soaking on MDA Content During Germination

**Figure 6:**
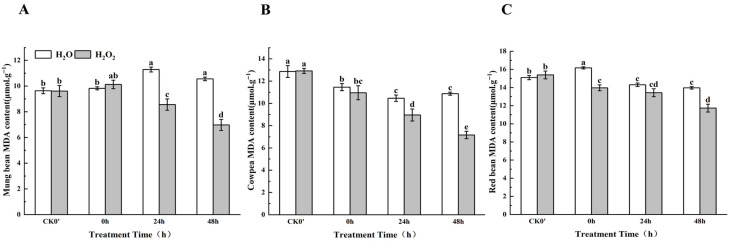
The MDA content in different legumes under H_2_O_2_ and water seed soaking treatment. (**A**) MDA content of mung beans. (**B**) MDA content of cowpeas. (**C**) MDA content of red beans. CK0′ represents the seeds at the end of soaking; “0 h” represents the seeds whose radicle has just broken through the seed coat (germination 0 h); “24 h” refers to seed samples which had been cultured for 24 h after germination; “48 h” refers to seed samples which had been cultured for 48 h after germination. The different letters mean significant differences at *p* < 0.05.

Malondialdehyde (MDA) is a byproduct of lipid peroxidation in plant biological membranes, and it reflects the extent of lipid peroxidation and cell damage within plants. The higher the level of stress a plant experiences, the higher the MDA content. As shown in Figure 6, compared to the control group, the MDA content in mung beans, cowpeas, and red beans decreased after H_2_O_2_ soaking, with the speed of decrease being significantly faster in the H_2_O_2_-treated group than in the control, indicating that H_2_O_2_ soaking reduced MDA content, thereby improving the stability of the cell membrane. Specifically, the MDA content in mung beans increased by 3.03% at 0 h of germination, and decreased by 24.14% and 36.36% at 24 h and 48 h, respectively, between the treatment group and the control group; this difference was most significant at 0 h and 24 h. In cowpeas, the MDA content decreased by 4.37%, 16.76%, and 29.62% at the same time points, with noticeable decreases at 24 h and 48 h. In red beans, the MDA content decreased by 13.60%, 6.13%, and 21.57% at 0 h, 24 h, and 48 h, between the treatment group and the control group; this difference was most significant at 0 h and 48 h. Based on this, it can be concluded that in terms of maintaining cell membrane stability, the H_2_O_2_ treatment was most effective on mung beans, followed by cowpeas, and least effective on red beans.

### 2.8. Effect of H_2_O_2_ Seed Soaking on POD Enzyme Activity During Seed Germination

**Figure 7:**
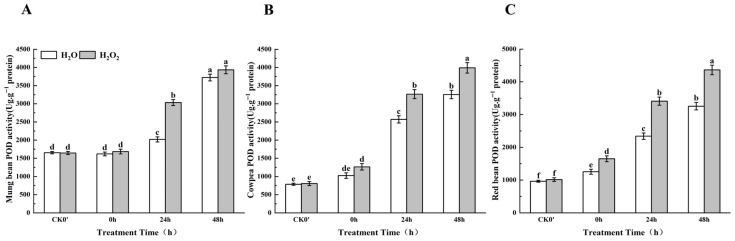
The POD enzyme activity in different legumes under H_2_O_2_ and water seed soaking treatment. (**A**) POD activity of mung beans. (**B**) POD activity of cowpeas. (**C**) POD activity of red beans. CK0′ represents the seeds at the end of soaking; “0 h” represents the seeds whose radicle has just broken through the seed coat (germination 0 h); “24 h” refers to seed samples which had been cultured for 24 h after germination; “48 h” refers to seed samples which had been cultured for 48 h after germination. The different letters mean significant differences at *p* < 0.05.

Plants respond to abiotic oxidative stress by modulating the activity of antioxidant enzymes. Compared to the control group, POD enzyme activity in the H_2_O_2_-treated seeds increased over the 0–48 h germination period (Figure 7). Specifically, in mung beans, POD activity increased by 3.85%, 33.38%, and 5.40% at 0 h, 24 h, and 48 h, respectively, between the treatment group and the control group; this difference was most significant at 24 h. In cowpea, POD activity increased by 18.97%, 21.28%, and 18.45% at the same time points, between the treatment group and the control group; this difference was most significant at both 24 h and 48 h. In red beans, POD activity increased significantly by 23.93%, 31.37%, and 25.40% at 0 h, 24 h, and 48 h, respectively. These results indicate that POD enzyme activity in all three legume species increased significantly after 24 h of germination, and the effects of H_2_O_2_ treatment on enzyme activity were most evident after this time point.

### 2.9. Effect of H_2_O_2_ Seed Soaking on SOD Enzyme Activity During Seed Germination

**Figure 8:**
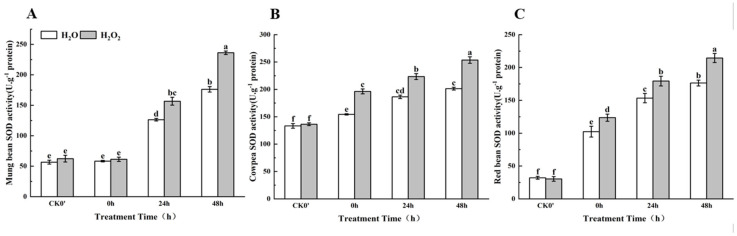
The SOD activity in different legumes under H_2_O_2_ and water seed soaking treatment. (**A**) SOD activity of mung beans. (**B**) SOD activity of cowpeas. (**C**) SOD activity of red beans. CK0′ represents the seeds at the end of soaking; “0 h” represents the seeds whose radicle has just broken through the seed coat (germination 0 h); “24 h” refers to seed samples which had been cultured for 24 h after germination; “48 h” refers to seed samples which had been cultured for 48 h after germination. The different letters mean significant differences at *p* < 0.05.

As shown in Figure 8, compared to the control group, SOD enzyme activity in the H_2_O_2_-treated seeds increased over the 0–48 h germination period. Specifically, in mung beans, SOD activity increased by 4.99%, 19.35%, and 25.36% at 0 h, 24 h, and 48 h, respectively, between the treatment group and the control group; this difference was most significant at both 24 h and 48 h. In cowpeas, SOD activity significantly increased by 21.28%, 16.56%, and 20.61% at 0 h, 24 h, and 48 h, respectively. In red beans, SOD activity increased by 17.21%, 14.49%, and 17.76% at 0 h, 24 h, and 48 h, respectively, with significant differences at all three time points. The results show that SOD enzyme activity in all three legumes increased over time. In cowpeas and red beans, SOD activity began to rise significantly after the radicle broke through the seed coat, and the H_2_O_2_ treatment had a noticeable effect on enzyme activity at this stage. In mung beans, however, SOD activity did not increase significantly until 24 h after the radicle emerged, and the effect of H_2_O_2_ treatment on SOD activity became more pronounced only after this time.

### 2.10. Effect of H_2_O_2_ Seed Soaking on CAT Enzyme Activity During Seed Germination

**Figure 9:**
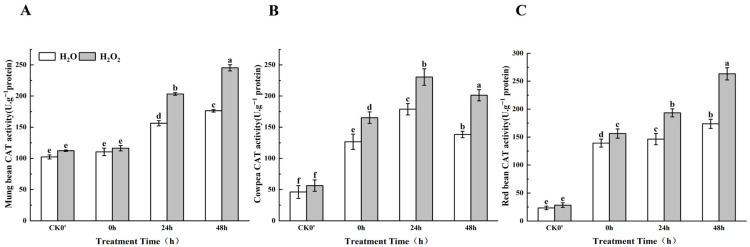
The CAT activity in different legumes under H_2_O_2_ and water seed soaking treatment. (**A**) CAT activity of mung beans. (**B**) CAT activity of cowpeas. (**C**) CAT activity of red beans. CK0′ represents the seeds at the end of soaking; “0 h” represents the seeds whose radicle has just broken through the seed coat (germination 0 h); “24 h” refers to seed samples which had been cultured for 24 h after germination; “48 h” refers to seed samples which had been cultured for 48 h after germination. The different letters mean significant differences at *p* < 0.05.

Although the CAT enzyme activity in both the control group and the H_2_O_2_-treated group showed an increasing trend, the increase in enzyme activity in the H_2_O_2_-treated group was significantly higher than that in the control group (Figure 9). Specifically, compared to the control group in mung beans, CAT enzyme activity increased by 5.12%, 23.12%, and 28.12% at 0 h, 24 h, and 48 h, respectively, with significant differences observed at 24 h and 48 h. In cowpeas, CAT enzyme activity significantly increased by 23.35%, 22.40%, and 31.25% at 0 h, 24 h, and 48 h, respectively. In red beans, CAT enzyme activity increased by 16.67%, 24.32%, and 34.03% at the same time points, between the treatment group and the control group; this difference was most significant at 24 h and 48 h. The results show that the increase in CAT enzyme activity mainly occurred after the radicle broke through the seed coat at 24 h. In mung beans and red beans, CAT enzyme activity increased over time, with the effect of H_2_O_2_ on enzyme activity becoming more pronounced after 24 h. In cowpeas, however, CAT enzyme activity showed an initial increase followed by a decrease, but the effect of H_2_O_2_ on enzyme activity was already evident after the radicle emerged from the seed coat.

### 2.11. Correlation Between Growth Parameters and Physiological Parameters of Mung Beans, Cowpeas, and Red Beans After H_2_O_2_ Soaking

**Figure 10:**
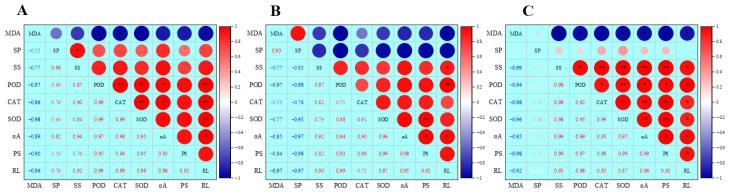
Correlation matrix of growth parameters and physiological parameters of mung beans, cowpeas, and red beans after H_2_O_2_ soaking. (**A**) Mung beans; (**B**) cowpeas; (**C**) red beans. MDA: malondialdehyde content; SP: soluble protein content; SS: soluble sugar content; CAT: catalase activity; POD: peroxidase activity; SOD: superoxide dismutase activity; αA: α-amylase activity; PS: protease activity; RL: radicle length; Red indicates a positive correlation, blue indicates a negative correlation, and the shade of the color represents the strength of the correlation. The numbers represent the Pearson correlation coefficient. * indicates a significant correlation at the 0.05 level and ** indicates a very significant correlation at the 0.01 level.

A correlation analysis was conducted on radicle length, MDA content, soluble sugar, soluble protein, and enzyme activity in mung beans, cowpeas, and red beans (Figure 10). In mung beans, radicle length showed an extremely significant positive correlation with POD, CAT, and SOD (*p* < 0.01), with correlation coefficients of 0.99; a significant positive correlation with α-amylase (*p* < 0.05), with a correlation coefficient of 0.98; and an extremely significant negative correlation with MDA (*p* < 0.01), with a correlation coefficient of 0.96. In cowpeas, radicle length showed an extremely significant positive correlation with POD (*p* < 0.01), with a correlation coefficient of 0.99; a significant positive correlation with soluble sugar and α-amylase (*p* < 0.05), with correlation coefficients of 0.90 and 0.95 respectively; and a significant negative correlation with MDA and soluble protein (*p* < 0.05), both with a correlation coefficient of 0.97. In red beans, radicle length showed a significant positive correlation with soluble sugar, POD, CAT, SOD, α-amylase, and protease (*p* < 0.05), with correlation coefficients of 0.93, 0.98, 0.85, 0.87, 0.96, and 0.92, respectively; and a significant negative correlation with MDA (*p* < 0.05), with a correlation coefficient of 0.92. The results showed that radicle growth in the three bean species was significantly positively correlated with antioxidant enzyme activity and the accumulation of soluble substances, but significantly negatively correlated with MDA content. These findings further demonstrate that hydrogen peroxide treatment promotes seed germination and early seedling growth in legumes by enhancing antioxidant capacity and facilitating the decomposition and transformation of storage materials.

## 3. Discussion

During seed germination, the synthesis and breakdown of storage substances provide energy for germination, growth, and other processes, forming an essential physiological foundation for plant development [18]. Leguminous crops are rich in nutrients such as starch and proteins, with starch making up approximately 50% and protein about 25% of the seed content [19,20]. During seed germination, the soluble sugar content in the three beans species gradually increased, indicating that starch in the seeds was being broken down into soluble sugars to provide nutrients and improve the rate for seed growth and development [21,22]. After H_2_O_2_ seed soaking, the soluble sugar content in the three species increased significantly, reaching up to 19.83%. However, the change in soluble sugar content varied among the species. In mung beans, the soluble sugar content began to decrease after 24 h of germination, which may be related to the nature of mung beans. Mung beans germinate faster than cowpeas and red beans. After soaking, the mung beans’ radicle immediately broke through the seed coat, while cowpeas and red beans had no signs of germination at that point. The decrease in soluble sugar in mung beans after 24 h may be due to the cessation of starch breakdown into soluble sugars, with soluble sugars being utilized as nutrients, resulting in a reduction in their content [23,24]. Soluble sugars also play a role in osmotic regulation [25,26]. After H_2_O_2_ seed soaking, seeds increased the breakdown of storage materials to raise the soluble sugar content, maintaining cellular osmotic pressure and preventing cell damage from excessive water absorption or dehydration [27,28]. At this stage, soluble sugars increase. However, after germination, as seedlings adapt to the environment, the soluble sugar content begins to decrease. Since mung beans germinate earlier, they adapt to the environment more quickly, and thus their soluble sugar content decreases after 24 h. Cowpeas and red beans, which germinate later and require more time to adapt, show a continuous increase in soluble sugar content from 0 to 48 h after germination.

The increase in α-amylase activity is crucial for improving germination performance. This enzyme is responsible for hydrolyzing starch into soluble sugars, providing the primary carbon source and energy for embryonic growth during the early stages of germination. In this study, all three legume species exhibited a significant increase in α-amylase activity 24 h after germination, indicating that hydrogen peroxide treatment accelerated starch degradation and sugar accumulation, thereby supporting faster radicle elongation and enhanced germination vigor. This result is consistent with the report by Jiang D et al. [29], which found that seed soaking treatment can increase α-amylase activity and improve plant germination capability under stress conditions.

Soluble protein content indirectly reflects the strength of seed metabolic activity [30]. In this study, mung beans and red beans showed the highest soluble protein content 24 h after germination, which may correspond to the peak of seed metabolic activity. As time passed, enzyme levels in the seeds decreased, and metabolic activity began to decline. In contrast, cowpeas showed the highest soluble protein content immediately after soaking, suggesting that enzymes in the seeds had already started to increase during the soaking process, thus storing sufficient nutrients for subsequent growth. The different trends in soluble protein content among the three species may be attributed to variations in physiological metabolism during germination. Moreover, soluble protein content is also influenced by stress. When plants experience stress, the soluble protein content typically decreases [31,32]. However, after H_2_O_2_ seed soaking, the soluble protein content in all three species significantly increased, with the highest increase reaching 10.85%, indicating that H_2_O_2_ treatment enhanced enzyme content, raised soluble protein levels, and improved water retention capacity in cells. This led to increased metabolic activity during growth, higher seed germination percentages, and improved seedling quality.

Protease activity is another crucial indicator of seed metabolic activation. Proteases catalyze the degradation of storage proteins into amino acids and peptides, which not only serve as nitrogen sources for embryo growth but also participate in the synthesis of new proteins essential for cell division and differentiation. The observed increase in protease activity in H_2_O_2_-treated seeds suggests that exogenous H_2_O_2_ promotes the mobilization of storage proteins and enhances metabolic activity during germination. Furthermore, the concurrent increase in soluble protein content and protease activity implies a dynamic balance between protein synthesis and degradation, which contributes to maintaining osmotic regulation and supporting embryo development.

Environmental stress is an unavoidable challenge during seed germination, and improving plant stress resistance is crucial for enhancing crop quality and yield [33,34]. Stress increases the level of reactive oxygen species (ROS) in plants, and the strength of antioxidant enzyme activity determines the plant’s ability to clear ROS [35,36,37]. Excessive ROS can not only cause the breakdown of cell membranes, producing more peroxidized lipid products, but also disrupt membrane integrity and lead to the release of phospholipids, resulting in structural damage. Damaged membrane systems cause a series of physiological and biochemical disorders, and prolonged or excessive stress can lead to plant death [27,38]. The main antioxidant enzymes in plants are POD, SOD, and CAT, each playing distinct roles. POD is mainly associated with plant respiration, photosynthesis, and other growth metabolic processes, and its activity reflects metabolic changes in the plant [39]. SOD catalyzes the dismutation of superoxide radicals into oxygen and H_2_O_2_, protecting cells and preventing membrane damage [40]. CAT removes superoxide anions and H_2_O_2_ to maintain normal plant growth [41,42]. This study found that after H_2_O_2_ seed soaking, the activities of POD, SOD, and CAT in mung beans, cowpeas, and red beans increased by 33.38%, 21.28%, and 34.03%, respectively. This suggests that H_2_O_2_ seed soaking enhances the seeds’ ability to scavenge ROS, reducing ROS-induced cellular damage, and significantly improving the resistance of the three beans species. The enhancement of antioxidant activity induced by H_2_O_2_ treatment may be intrinsically related to the efficiency with which stored energy substances in seeds are utilized. We speculate that an activated, efficient antioxidant system indirectly promotes or protects the activity of key enzymes involved in germination by maintaining cellular redox homeostasis, thereby accelerating the germination process of legume seeds. Under stress conditions, plants often undergo membrane lipid peroxidation, and malondialdehyde (MDA) is the final product of this process. Its content is closely related to the degree of stress-induced damage in plants [43]. This study showed that after H_2_O_2_ seed soaking, the MDA content decreased significantly in all three species, with reductions of 36.36%, 29.62%, and 21.57%, respectively, indicating that H_2_O_2_ treatment reduced membrane lipid degradation, increased cell membrane stability, and enhanced stress resistance.

## 4. Materials and Methods

### 4.1. Seed Materials and Treatments

The seeds of mung beans (Keda Green No. 2), cowpeas (Keda Cowpea No. 1), and red beans (Jihong 352) were disinfected with 0.5% sodium hypochlorite solution for 3 min, followed by three washes with sterile water. After disinfection, the seeds were soaked in either distilled water or a 50 mmol/L H_2_O_2_ solution for 12 h at 25 °C, with distilled water serving as the control. After soaking, 30 seeds from each legume were placed in 15 cm diameter Petri dishes, each lined with two layers of filter paper, moistened adequately. The experiment was repeated three times, and the seeds were incubated at 25 °C in the dark for 5 days. During this period, the filter paper was watered once daily to maintain sufficient moisture. The following measurements were taken:

### 4.2. Seed Germination Indices Measurement

During seed germination, germination energy percentage was measured on day 1, germination percentage on day 3, and germination vigor percentage on day 5. The germination index was also calculated. Germination was defined as the moment the radicle broke through the seed coat.

Germination energy percentage = (Number of seeds with radicle ≥ 3 mm on day 1/Total number of seeds) × 100%

Germination percentage = (Number of seeds with radicle ≥ 3 mm on day 3/Total number of seeds) × 100%

Germination index = Σ(Gt/Dt)

where Gt is the number of germinated seeds on day t, and Dt is the corresponding germination day.

Radicle Length Measurement: At 24 h, 48 h, and 72 h after germination, three seedlings from each treatment were randomly selected. Surface moisture was absorbed with filter paper, and the radicle length was measured using a vernier caliper.

### 4.3. Biochemical Measurements

Sampling procedure: Samples were collected at 0 h, 24 h, and 48 h of germination. For each treatment and time point, three uniformly developed seedlings were randomly selected, and the entire seedlings (including radicle, hypocotyl, plumule, and cotyledon parts) were taken as the test material and mixed to form one biological replicate. The samples were immediately frozen in liquid nitrogen and stored at −80 °C. The samples were then subjected to cryogenic grinding to obtain a fine powder. Each treatment consisted of three biological replicates, and each biochemical assay was performed with three technical replicates to ensure data accuracy. Samples were collected at 0 h, 24 h, and 48 h of germination at 25 °C and immediately frozen in liquid nitrogen. They were then stored at −80 °C in an ultra-low temperature freezer.

Soluble sugar content was measured using the anthrone method and standardized with sucrose. The sample was boiled, then cooled using a mixture of ultrapure water and anthrone–sulfuric acid, and the absorbance was measured at 620 nm [44].

α-amylase activity was measured using the 3, 5-dinitrosalicylic acid (DNS) method [45]. Take 0.1 g of the ground sample into a 1.5 mL centrifuge tube, add 1 mL of distilled water, and extract thoroughly at room temperature for 15–20 min. Centrifuge at 3000 r/min for 10 min, then decant the entire supernatant and dilute to a final volume of 10 mL. Subsequent steps were performed according to the method described by Nauman M K et al., with colorimetric measurement conducted at 540 nm using a spectrophotometer (T6 New Century, Puxi General Instruments Company, Beijing, China).

Protease activity: Using tyrosine as the standard, the absorbance at 275 nm was measured, and the results are expressed in U·g^−1^ [46].

Soluble protein content was measured using the Coomassie Brilliant Blue colorimetric method [47]. Take 0.1 g of the sample, add 1 mL of distilled water, and centrifuge at 4 °C and 4000 r/min for 10 min. Then, take 50 μL of the supernatant, add 3 mL of Coomassie Brilliant Blue G-250 solution, and let it stand for 2 min. Perform colorimetric measurement at 595 nm using a UV–visible spectrophotometer, with zero adjustment set using a mixture of 50 μL of the extraction buffer and 3 mL of Coomassie Brilliant Blue G-250 solution.

Malondialdehyde (MDA) content was measured using the thiobarbituric acid (TBA) method [47]. A 0.5 g sample was homogenized with 5 mL of 5% thiobarbituric acid (TBA) and centrifuged at 3000 r/min for 10 min. Then, 2 mL of the supernatant was mixed with 2 mL of 0.67% TBA. The mixture was heated in a boiling water bath at 100 °C for 30 min, cooled, and centrifuged again. The absorbance of the resulting supernatant was measured at 450 nm, 532 nm, and 600 nm. The MDA concentration was calculated based on these values and expressed as μ mol per gram of fresh weight tissue (μ mol/g).

Enzyme extraction: Take 0.1 g of the sample and add 1 mL of phosphate buffer (pH 7.8) into a 1.5 mL centrifuge tube. Keep the tube on ice, then centrifuge under refrigeration for 20 min. Transfer the supernatant (enzyme extract) into a test tube and store at 0–4 °C for subsequent use.

Peroxidase (POD) activity was measured using the guaiacol–trichloroacetic acid (TCA) method [48]. Take 0.1 mL of the enzyme solution and add it to 2.9 mL of the POD reaction solution. Measure the absorbance at 470 nm using a spectrophotometer at 1 min intervals, recording a total of three measurements. The enzyme activity is expressed as the change in absorbance per minute.

Catalase (CAT) activity was measured using the hydrogen peroxide reduction method [48]. Take 0.2 mL of the enzyme solution and add it to 2.8 mL of the CAT reaction solution. Measure the absorbance at 240 nm using a spectrophotometer at 30 s intervals for a total duration of 3 min. The enzyme activity is expressed as the change in absorbance per minute.

Superoxide dismutase (SOD) activity was measured using the nitroblue tetrazolium (NBT) photoreduction method [48]. Take 0.1 mL of the enzyme solution and add it to 3 mL of the SOD reaction solution. Prepare three replicates for the control tube, and for the blank tube, replace the enzyme solution with buffer. Place the blank tube in a dark environment, while exposing the control and test tubes to 4000 r/min for 30 min. Use the blank tube to zero the instrument, and perform colorimetric measurement at 560 nm using a UV–visible spectrophotometer.

### 4.4. Data Analysis

Data were analyzed using SPSS software (Version 26.0; SPSS Inc., Chicago, IL, USA) for one-way analysis of variance (ANOVA), with the least significant difference (LSD) test (*p* < 0.05) used to compare differences between samples. Graphs were created using Origin 2021 (Origin Lab Corporation, Northampton, MA, USA) and Microsoft Excel 2016 (Microsoft Corporation, Redmond, WA, USA) for data visualization. All parameters were determined with at least three replicates.

## 5. Conclusions

In conclusion, soaking seeds in a 50 mmol/L H_2_O_2_ solution effectively enhances the germination percentage on day 1 and day 3, the soluble sugar content, and the soluble protein content of mung bean, cowpea, and red bean seeds. Exogenous hydrogen peroxide also increases antioxidant enzyme activity, α-amylase activity, and protease activity; reduces malondialdehyde (MDA) content; and significantly increases the radicle length of treated seeds compared to untreated seeds. Additionally, seedling cotyledon growth is significantly accelerated. These results suggest that H_2_O_2_ promotes seed germination by influencing the germination percentage, the antioxidant enzyme activity, the breakdown of storage substances, and the regulation of substances involved in germination growth, thereby enhancing stress resistance during the seed germination process. These findings provide a theoretical basis for improving seed germination percentage and seedling stress resistance. However, the mechanisms by which H_2_O_2_ regulates the metabolic processes of nutrients during germination in mung beans, cowpeas, and red beans, and the key processes involved, such as enzyme activity and gene expression, require further research.

## Figures and Tables

**Table 1:** Effects of H_2_O_2_ soaking on the germination of mung beans, cowpeas, and red beans.

Treatment	Germination Energy Percentage (%)	Germination Percentage (%)	Germination Vigor Percentage (%)	Germination Index
GBCK	14.44 ± 1.73 d	67.78 ± 4.35 f	98.89 ± 0.58 a	46.67 ± 6.23 d
GBH	63.33 ± 4.58 a	98.89 ± 0.58 a	98.96 ± 0.58 a	59.58 ± 4.35 a
CBCK	13.33 ± 2.64 e	72.22 ± 3.00 e	81.11 ± 1.15 d	40.93 ± 5.70 e
CBH	34.44 ± 1.52 b	96.67 ± 1.15 b	96.67 ± 0.58 b	56.87 ± 6.85 b
RBCK	0.00 ± 0.00 f	75.56 ± 2.64 d	76.66 ± 1.52 e	27.48 ± 2.56 f
RBH	18.89 ± 2.51 c	93.33 ± 2.08 c	94.44 ± 2.08 c	52.67 ± 4.58 c

GBCK: Mung bean control check. GBH: Mung beans treated with H_2_O_2_. CBCK: Cowpea control check. CBH: Cowpeas treated with H_2_O_2_. RBCK: Red bean control check. RBH: Red beans treated with H_2_O_2_. The values in the table are mean ± standard deviation, and different lowercase letters for the same indicator indicate significant differences among treatments (*p* < 0.05).

**Table 2:** Effects of H_2_O_2_ soaking on the radicle length of mung beans, cowpeas, and red beans.

Treatment	24 h Radicle Length (cm)	48 h Radicle Length (cm)	72 h Radicle Length (cm)
GBCK	1.72 ± 0.27 d	2.83 ± 0.23 d	5.38 ± 0.19 b
GBH	3.23 ± 0.24 a	4.54 ± 0.24 a	7.28 ± 0.19 a
CBCK	2.14 ± 0.24 c	3.13 ± 0.13 c	3.83 ± 0.32 e
CBH	2.83 ± 0.13 b	4.23 ± 0.12 b	5.22 ± 0.17 c
RBCK	0.93 ± 0.13 f	2.00 ± 0.18 f	3.38 ± 0.23 f
RBH	1.55 ± 0.14 e	2.48 ± 0.13 e	4.35 ± 0.22 d

GBCK: Mung bean control check. GBH: Mung beans treated with H_2_O_2_. CBCK: Cowpea control check. CBH: Cowpeas treated with H_2_O_2_. RBCK: Red bean control check. RBH: Red beans treated with H_2_O_2_. The values in the table are mean ± standard deviation, and different lowercase letters for the same indicator indicate significant differences among treatments (*p* < 0.05).

## Data Availability

The original contributions presented in this study are included in the article. Further inquiries can be directed to the corresponding authors.

## References

[1] Ajayakumar, Patil S.S., Reddy S.B., Goudappa S.B. (2024). Preference Analysis of Pigeonpea Varietal Attributes among Farmers and Traders in Kalyana Karnataka Region: A Conjoint Analysis Approach. Asian J. Agric. Ext. Econ. Sociol..

[2] Saulle C.C., Claus A., Sales L.D.A., Gonçalves A.G., Ducatti D.R., Noseda M.D., May De Mio L.L. (2022). Photoinactivation of *Colletotrichum truncatum*, *Corynespora cassiicola*, *Sclerotinia sclerotiorum* and *Rhizoctonia solani* in soybean seeds by cationic porphyrins. Plant Pathol..

[3] Rakavi B., Sridhar S., Srimahesvari D.S., Subashree A., Suganya S., Sujitha P., SuryaRishi S. (2022). Physiological Screening of Green Gram (*Vigna radiata* L.) Varieties by Seedling Germination Traits under PEG Induced Drought Stress. Int. J. Plant Soil Sci..

[4] Peñaranda I., Garrido D.M., Segovia G.P. (2025). Effect of protein texturization on amino acids and protein in vitro bio-accessibility of pea and rice protein. Food Funct..

[5] Peñaranda I., Garrido M.D., García-Segovia P., Martínez-Monzó J., Igual M. (2025). Dry Fractionation for Sustainable Production of Functional, Nutritional and Palatable Grain Legume Protein Ingredients. Food Eng. Rev..

[6] Spanic V., Duvnjak J., Hefer D. (2024). Changes in Metabolites Produced in Wheat Plants Against Water-Deficit Stress. Plants.

[7] Spanic V., Duvnjak J., Hefer D., D’Auria J.C. (2023). Effect of Abiotic Stresses from Drought, Temperature, and Density on Germination and Seedling Growth of Barley (*Hordeum vulgare* L.). Plants.

[8] Sun X.M., Wang S.S., Yang H.G. (2015). Antioxidant Enzymes Activity of *Paeonia lactiflora* During Seed Germination. North. Hortic..

[9] Chen X.F., Zhang M., Li B., Cui T., Liu C., Liu C.J., Chen B.R., Zhou Y.F. (2023). CaCl_2_ priming promotes sorghum seed germination under salt stress by activating sugar metabolism. Plant Growth Regul. Int. J. Nat. Synth. Regul..

[10] Zhao X., Ma K., Li Z., Li W., Zhang X., Liu S., Yuan X. (2023). Transcriptome Analysis Reveals Brassinolide Signaling Pathway Control of Foxtail Millet Seedling Starch and Sucrose Metabolism under Freezing Stress, with Implications for Growth and Development. Int. J. Mol. Sci..

[11] Wang J., Yan D., Liu R., Wang T., Lian Y., Lu Z., Li R. (2024). The Physiological and Molecular Mechanisms of Exogenous Melatonin Promote the Seed Germination of Maize (*Zea mays* L.) under Salt Stress. Plants.

[12] Ou Y., Teng Z., Shu Y., Wang Y., Wang D., Sun C., Lin X. (2025). Linoleic acid alleviates aluminum toxicity by modulating fatty acid composition and redox homeostasis in wheat (*Triticum aestivum*) seedlings. J. Hazard. Mater..

[13] Yan L., Liu S., Li R., Li Z., Piao J., Zhou R. (2024). Calcium enhanced the resistance against *Phoma arachidicola* by improving cell membrane stability and regulating reactive oxygen species metabolism in peanut. BMC Plant Biol..

[14] Kaur R., Gupta K.A., Taggar K.G. (2014). Nitrate reductase and nitrite as additional components of defense system in pigeonpea (*Cajanus cajan* L.) against *Helicoverpa armigera* herbivory. Pestic. Biochem. Physiol..

[15] Bilal Hafeez M., Ghaffar A., Zahra N., Ahmad N., Raza A., Wang R., Li J. (2024). Effect of Plant Growth Regulators on Water Relations, Proximate Composition, and Ascorbate/glutathione Cycle of Late-sown Wheat Under Saline Conditions. J. Crop Health.

[16] Wang Y., Liu X., Sun X., Mao X., Wang Z., Peng J., Li F. (2025). The promotive and repressive effects of exogenous H_2_O_2_ on Arabidopsis seed germination and seedling establishment depend on application dose. Physiol. Plant..

[17] Muñoz-Salinas F., Tovar-Pérez E.G., Guevara-González R.G., Loarca-Piña G.F., Torres-Pacheco I. (2021). Effect of Hydrogen Peroxide Pretreatment on Physiological and Biochemical Variables during Germination of Alfalfa Seeds. Legume Res.-Int. J..

[18] Verma J.P., Yadav J., Tiwari K.N., Kumar A. (2013). Effect of indigenous *Mesorhizobium* spp. and plant growth promoting rhizobacteria on yields and nutrients uptake of chickpea (*Cicer arietinum* L.) under sustainable agriculture. Ecol. Eng..

[19] Springmann M. (2024). A multicriteria analysis of meat and milk alternatives from nutritional, health, environmental, and cost perspectives. Proc. Natl. Acad. Sci. USA.

[20] Rotundo J.L., Marshall R., McCormick R., Truong S.K., Styles D., Gerde J.A., Rufino M.C. (2024). European soybean to benefit people and the environment. Sci. Rep..

[21] Huang Y., Mei G., Cao D., Qin Y., Yang L., Ruan X. (2023). Spermidine enhances heat tolerance of rice seeds during mid-filling stage and promote subsequent seed germination. Front. Plant Sci..

[22] Wang Y., Yan H., Ma T., Li F., Liu Z. (2019). Study on germination and the content of proline, soluble sugar, starch, fat and soluble protein of *Idesia Polycarpa* Maxim. seed at low temperature. FEB-Fresenius Environ. Bull..

[23] Wang H., Xu T., Li Y., Gao R., Tao X., Song J., Li Q. (2024). Comparative transcriptome analysis reveals the potential mechanism of GA3-induced dormancy release in *Suaeda glauca* black seeds. Front. Plant Sci..

[24] Radhakrishnan R., Kumari B.D.R. (2013). Influence of pulsed magnetic field on soybean (*Glycine max* L.) seed germinate seedling growth and soil microbial population. Indian J. Biochem. Biophys..

[25] Cheng Y., Cao M., Shi X., Chen X., Li Z., Ma Y. (2025). Mitigating salt stress in *Zea mays*: Harnessing *Serratia nematodiphila*-biochar-based seed coating for plant growth promotion and rhizosphere microecology regulation. Ind. Crops Prod..

[26] Chen L., Liu L.T., Lu B., Ma T.T., Jiang D., Li J., Zhang K., Sun H.C., Zhang Y.J., Bai Z.Y. (2020). Exogenous melatonin promotes seed germination and osmotic regulation under salt stress in cotton (*Gossypium hirsutum* L.). PLoS ONE.

[27] El-Shazoly R.M., Othman A.A., Zaheer M.S., Al-Hossainy A.F., Abdel-Wahab D.A. (2025). Zinc oxide seed priming enhances drought tolerance in wheat seedlings by improving antioxidant activity and osmoprotection. Sci. Rep..

[28] El-Shazoly R.M., Othman A.A., Zaheer M.S., Al-Hossainy A.F., Abdel-Wahab D.A. (2025). Nitric Oxide and Hydrogen Peroxide Coordinate to Improve Photosynthesis, Oxidative Defense, Osmoregulation, and Ions Homeostasis in Pea (*Pisum sativum* L.) Under Drought. J. Soil Sci. Plant Nutr..

[29] Jiang D.X., Ou Y., Jiang G.C., Dai G., Liu S.H., Chen G.X. (2025). Melatonin-priming ameliorates aluminum accumulation and toxicity in rice through enhancing aluminum exclusion and maintaining redox homeostasis. Plant Physiol. Biochem..

[30] Su X., Wang X., Cui N., Xu H., Tian T., Wei B. (2025). Enhancing germination and growth in *Malania oleifera* Chun & SK Lee seeds through gibberellic acid priming. J. Appl. Res. Med. Aromat. Plants.

[31] Jan M.F., Altaf M.T., Liaqat W., Liu C., Mohamed H.I., Li M. (2025). Approaches for the amelioration of adverse effects of drought stress on soybean plants: From physiological responses to agronomical, molecular, and cutting-edge technologies. Plant Soil.

[32] Xu Q., Yan Y., Wei Q., Wang H., Chi C., Pan L., Zhu C. (2025). Salicylic acid alleviates cold stress in Rice via regulating nutrient absorption, osmotic material content, Antioxidation System, and expression of Cold Tolerance genes. J. Plant Growth Regul..

[33] Priya M., Farooq M., Siddique K.H.M. (2025). Enhancing Tolerance to Combined Heat and Drought Stress in Cool-Season Grain Legumes: Mechanisms, Genetic Insights, and Future Directions. Plant Cell Environ..

[34] Rao M.J., Zheng B. (2025). The Role of Polyphenols in Abiotic Stress Tolerance and Their Antioxidant Properties to Scavenge Reactive Oxygen Species and Free Radicals. Antioxidants.

[35] Ashraf H., Ghouri F., Ali S., Bukhari S.A.H., Haider F.U., Zhong M., Shahid M.Q. (2025). The protective roles of Oryza glumaepatula and phytohormone in enhancing rice tolerance to cadmium stress by regulating gene expression, morphological, physiological, and antioxidant defense system. Environ. Pollut..

[36] Tang Y., Ding Y., Nadeem M., Li Y., Zhao W., Guo Z., Rui Y. (2025). Enhancing maize stress tolerance with nickel ferrite nanoparticles: A sustainable approach to combat abiotic stresses. Environ. Sci. Nano.

[37] Rahman M.A., Lee S.H., Park H.S., Min C.W., Woo J.H., Choi B.R., Lee K.W. (2025). Light Quality Plays a Crucial Role in Regulating Germination, Photosynthetic Efficiency, Plant Development, Reactive Oxygen Species Production, Antioxidant Enzyme Activity, and Nutrient Acquisition in Alfalfa. Int. J. Mol. Sci..

[38] Aswathi K.P.R., Jisha K.C., Veena M., Sen A., Sarath N.G., Puthur J.T. (2025). GABA Priming Induced Modulations in the Redox Homeostasis of Plants under Osmotic Stress. GABA in Plants: Biosynthesis, Plant Development, and Food Security.

[39] Liu N. (2014). Antioxidant Enzymes Regulate Reactive Oxygen Species during Pod Elongation in *Pisum sativum* and *Brassica chinensis*. PLoS ONE.

[40] Rezgui M., Ammar W.B., Nazim M., Soufan W., Haouari C.C. (2025). Exogenous Alpha-Ketoglutarate (AKG) Modulate Physiological Characteristics, Photosynthesis, Secondary Metabolism and Antioxidant Defense System in *Peganum Harmala* L. under Nickel Stress. Phyton-Int. J. Exp. Bot..

[41] Wu Y., Zhang L., Zhang Y., Zhou H.W., Ma L. (2024). Roles of Antioxidant Enzymes, Secondary Metabolites, and Lipids in Light Adaption of Tea-Oil Plant (*Camellia oleifera* Abel). J. Plant Growth Regul..

[42] Wang H., Zhang Y., Jiang H., Ding Q., Wang Y., Wang M., Jia L. (2025). Transcriptomic and metabolomic analysis reveals the molecular mechanism of exogenous melatonin improves salt tolerance in eggplants. Front. Plant Sci..

[43] Lamlom S.F., Abdelghany A.M., Farouk A.S., Alwakel E.S., Makled K.M., Bukhari N.A., Shehab A.A. (2025). Biochemical and yield response of spring wheat to drought stress through gibberellic and abscisic acids. BMC Plant Biol..

[44] Liang Y., Chen Q., Liu Q., Zhang W., Ding R. (2003). Exogenous silicon (Si) increases antioxidant enzyme activity and reduces lipid peroxidation in roots of salt-stressed barley (*Hordeum vulgare* L.). J. Plant Physiol..

[45] Nauman M.K., Li Y.H., Zaid K., Chen L.L., Liu J.H., Hu J., Wu H.H., Li Z.H. (2021). Nanoceria seed priming enhanced salt tolerance in rapeseed through modulating ROS homeostasis and α-amylase activities. J. Nanobiotechnol..

[46] Guo X., Zhou H., Yu Z., Zhang Y. (2007). Changes in the distribution of nitrogen and plant enzymatic activity during ensilage of lucerne treated with different additives. Grass Forage Sci..

[47] Bradford M.M. (1976). A rapid and sensitive method for the quantitation of microgram quantities of protein utilizing the principle of protein-dye binding. Anal. Biochem..

[48] Fu J., Huang B. (2001). Involvement of antioxidants and lipid peroxidation in the adaptation of two cool-season grasses to localized drought stress. Environ. Exp. Bot..

